# Prognostic and Clinical Value of Cluster Analysis in Idiopathic Pleuroparenchymal Fibroelastosis Phenotypes

**DOI:** 10.3390/jcm10071498

**Published:** 2021-04-04

**Authors:** Yutaro Nakamura, Kazutaka Mori, Yasunori Enomoto, Masato Kono, Hiromitsu Sumikawa, Takeshi Johkoh, Thomas V. Colby, Hideki Yasui, Hironao Hozumi, Masato Karayama, Yuzo Suzuki, Kazuki Furuhashi, Tomoyuki Fujisawa, Noriyuki Enomoto, Naoki Inui, Yusuke Kaida, Koshi Yokomura, Naoki Koshimizu, Mikio Toyoshima, Shiro Imokawa, Takashi Yamada, Toshihiro Shirai, Hidenori Nakamura, Hiroshi Hayakawa, Takafumi Suda

**Affiliations:** 1 Second Division, Department of Internal Medicine, Hamamatsu University School of Medicine, Shizuoka 431-3192, Japan; enomotoy@hama-med.ac.jp (Y.E.); yasui@hama-med.ac.jp (H.Y.); hozumi@hama-med.ac.jp (H.H.); karayama@hama-med.ac.jp (M.K.); yuzosuzu@hama-med.ac.jp (Y.S.); furuhashi@hama-med.ac.jp (K.F.); fujisawa@hama-med.ac.jp (T.F.); norieno@hama-med.ac.jp (N.E.); inui@hama-med.ac.jp (N.I.); suda@hama-med.ac.jp (T.S.); 2 Department of Internal Medicine, Shizuoka City Shimizu Hospital, Shizuoka 424-8636, Japan; mori-kazutaka@shimizuhospital.com; 3 Department of Respiratory Medicine, Seirei Hamamatsu General Hospital, Shizuoka 430-8558, Japan; masato.k@sis.seirei.or.jp (M.K.); hnakamura@sis.seirei.or.jp (H.N.); 4 Department of Radiology, Sakai City Medical Center, Osaka 593-8304, Japan; h-sumikawa@radiol.med.osaka-u.ac.jp; 5 Department of Diagnostic Radiology, Kansai Rosai Hospital, Hyogo 660-8511, Japan; johkoht@gmail.com; 6 Department of Laboratory Medicine and Pathology (Emeritus), Mayo Clinic Arizona, Scottsdale, AZ 85259, USA; colby.thomas@mayo.edu; 7 Department of Clinical Pharmacology and Therapeutics, Hamamatsu University School of Medicine, Shizuoka 431-3192, Japan; 8 Department of Internal Medicine, Enshu Hospital, Shizuoka 430-0929, Japan; ykaida@gmail.com; 9 Department of Respiratory Medicine, Seirei Mikatahara General Hospital, Shizuoka 433-8558, Japan; yo.koshi@sis.seirei.or.jp; 10 Department of Respiratory Medicine, Fujieda Municipal General Hospital, Shizuoka 426-8677, Japan; pineapplefish1219@yahoo.co.jp; 11 Department of Respiratory Medicine, Hamamatsu Rosai Hospital, Shizuoka 430-8525, Japan; mi-toyoshima@hamamatsuh.johas.go.jp; 12 Department of Respiratory Medicine, Iwata City Hospital, Shizuoka 438-8550, Japan; imokawas@hospital.iwata.shizuoka.jp; 13 Department of Respiratory Medicine, Shizuoka City Shizuoka Hospital, Shizuoka 426-8630, Japan; bxh03346@nifty.com; 14 Department of Respiratory Medicine, Shizuoka General Hospital, Shizuoka 420-8527, Japan; toshihiro-shirai@i.shizuoka-pho.jp; 15 Department of Respiratory Medicine, Tenryu Hospital, National Hospital Organization, Shizuoka 434-8511, Japan; hayakawa.hiroshi.rz@mail.hosp.go.jp

**Keywords:** idiopathic pleuroparenchymal fibroelastosis, cluster analysis, prognosis

## Abstract

Idiopathic pleuroparenchymal fibroelastosis (PPFE) is a distinctive interstitial pneumonia with upper lobe predominance that shows unique morphological features among idiopathic interstitial pneumonias (IIPs). Affected patients have a variety of clinical presentations with heterogeneous clinical courses. Cluster analysis is a valuable tool for identifying distinct clinical phenotypes under heterogeneous conditions. This study aimed to identify the phenotypes of patients with idiopathic PPFE. Using cluster analysis, novel PPFE phenotypes were identified among subjects from our multicenter cohort, and outcomes were stratified according to phenotypic clusters. Among the subjects with baseline data (*N* = 84), four clusters were identified. Cluster 1 included younger male subjects with coexisting non-UIP-like patterns. Cluster 2 included elderly female nonsmokers with low body mass index (BMI). Cluster 3 included elderly male smokers with a coexisting IP-like pattern. Cluster 4 included younger male smokers without lower lobe lesions. Patients in cluster 3 had significantly worse survival outcomes than those in clusters 1, 2, and 4 (*p* < 0.001, *p* = 0.0041, and *p* = 0.0155, respectively). Among idiopathic PPFE patients, cluster analysis using baseline characteristics identified four distinct clinical phenotypes that might predict survival outcomes.

## 1. Introduction

Idiopathic pleuroparenchymal fibroelastosis (PPFE) is a rare disorder that includes idiopathic interstitial pneumonias (IIPs) according to the updated American Thoracic Society/European Respiratory Society classification [1], characterized by predominantly upper lobe pleural and subjacent parenchymal fibrosis [2]. However, it has been reported that patients with idiopathic PPFE have various clinical presentations with a heterogeneous clinical course [3].

Cluster analysis modeling is a method for identifying distinct clinical phenotypes under heterogeneous conditions [4,5,6]. No study has applied cluster analysis to identify heterogeneity in patients with idiopathic PPFE. Therefore, this study aimed to identify phenotypes using cluster analysis in idiopathic PPFE and to compare differences in clinical, physiological, radiological, and survival data among the clusters.

## 2. Materials and Methods

### 2.1. Patients and Diagnostic Criteria for Idiopathic PPFE

This retrospective multicenter study included patients diagnosed with PPFE or idiopathic pulmonary upper lobe fibrosis (IPUF), a term formerly applied to some cases of PPFE, who were admitted to Hamamatsu University School of Medicine and the nine associated hospitals from 2005 to 2016. Patients were consecutively recruited for the study. The diagnosis of idiopathic PPFE was based on the following criteria [7]: (1) a radiologic PPFE pattern on chest computed tomography (CT) characterized as bilateral subpleural dense consolidation with or without pleural thickening in the upper lobes and less marked or absent involvement of the lower lobes; (2) radiologic confirmation of disease progression, which was characterized by an increase in the upper lobe consolidation with or without pleural thickening and/or a decrease in upper lobe volume on serial radiologic assessment; and (3) exclusion of other lung diseases with identifiable etiology, such as connective tissue disease (CTD), chronic hypersensitivity pneumonitis (CHP), pulmonary sarcoidosis, pneumoconiosis, and active pulmonary infection. Chest CT images were reviewed independently by two expert chest radiologists with 31 and 15 years of experience, respectively. Radiologic interstitial lung disease (ILD) patterns in patients with lower-lobe ILD were classified as either usual interstitial pneumonia (UIP), probable UIP, indeterminate for UIP, or alternative diagnosis based on the criteria mentioned in an official clinical practice guideline of idiopathic pulmonary fibrosis (IPF) by the American Thoracic Society (ATS)/European Respiratory Society (ERS)/Japanese Respiratory Society/Latin American Thoracic Association [8]. In this study, the ‘UIP-like’ pattern includes UIP, probable UIP, and indeterminate for UIP patterns. The histological criteria for PPFE [2] were applied to patients whose lung specimens were obtained. The slides were reviewed by local pathologists and experienced lung pathologists.

### 2.2. Data Collection

Twenty-three variables were identified from each patient’s records with substantial clinical relevance for inclusion in the cluster analysis model based on previous literature [4,9]. The variables were as follows: demographic information (age, sex, body mass index [BMI]), patient-reported historical information (tobacco use and other environmental exposures [organic or inorganic]), comorbid disease conditions (gastroesophageal disease, malignancy, pneumothorax), symptoms (cough, dyspnea), physical examination findings (crackles), laboratory studies (antinuclear antibody [ANA] titer [>320 diffuse, speckled, homogeneous patterns or nucleolar pattern (any titer) or centromere pattern (any titer)], positive rheumatoid factor [>2, the upper limit of normal], other positive autoantibodies in the serological domain of interstitial pneumonia with autoimmune features (IPAF) criteria [10], serum albumin, LDH, sialylated carbohydrate antigen Krebs von den Lungen-6 [KL-6], surfactant protein [SP]-D, PaO2, PaCO2), pulmonary function tests (PFTs) (FVC, FEV1/FVC, and diffusion capacity of the lung for carbon monoxide [DLCO], residual volume/total lung capacity [RV/TLC]), and HRCT imaging findings. Survival and outcome data were also analyzed.

### 2.3. Statistical Analysis

The partitioning around medoids (PAM) clustering algorithm was used to cluster ILD subjects into groups with similar clinical phenotypes based on the 23 baseline variables. The fundamental principle underlying cluster analysis aims to classify subjects based on pre-specified variables to optimize cluster homogeneity and differentiate clusters from one another. PAM cluster analysis was performed to minimize the dissimilarity of each cluster and the use of medoids, which are the subjects in the dataset representative of each cluster. This method is similar to the previous method; however, it is more robust to outliers than the commonly used *k*-means clustering algorithm because of its reliability on medians than means. Continuous and categorical variables are included in the algorithm. As a result, the variables were scaled using Gower’s distance. The variables were scaled from zero to one prior to clustering. To determine the optimal number of clusters, the silhouette width was used to measure the similarity of a patient to his or her assigned cluster compared to neighboring clusters. PAM cluster analysis was performed using the ‘cluster’ package in R (R Foundation for Statistical Computing, Vienna, Austria). Continuous variables are reported as mean ± standard deviation, and categorical variables are reported as counts and percentages. Survival was assessed using unadjusted log-rank testing along with univariate and multivariable Cox proportional hazards regression. Survival curves were plotted using the Kaplan–Meier survival estimator. In multiple comparisons of demographic and clinical differences among the identified clusters, a Kruskal-Wallis rank-sum test was used for ordinal and continuous variables, and Fisher’s exact test was used for categorical variables.

## 3. Results

### 3.1. Subject Demographic Characteristics

In total, 109 patients were diagnosed based on the criteria, and 25 patients were excluded: 15 had other lung diseases (CTD, *n* = 9; active pulmonary infection, *n* = 4; CHP, *n* = 1; pneumoconiosis, *n* = 1), eight patients had no confirmed disease progression radiologically, and two had inadequate clinical information. The 84 remaining patients with confirmed idiopathic PPFE were enrolled in the study. The mean age of the patients was 68 years. More than 60% of the patients were male, one-third were smokers, and their body mass index (BMI) was 17.0 (low). The serum surfactant protein-D (SP-D) level was significantly higher than the normal range (231.0). The mean forced vital capacity (FVC) was 61.7%, and the diffusing capacity of the lung for carbon monoxide (DLCO) was 77.6%. Meanwhile, the residual volume/total lung capacity was predicted to be 48.8% (Table 1).

The data for the other variables are shown in the Supplementary Table S1. There was a small number of biopsy-proven cases (eight surgical lung biopsies).

**Figure 1:**
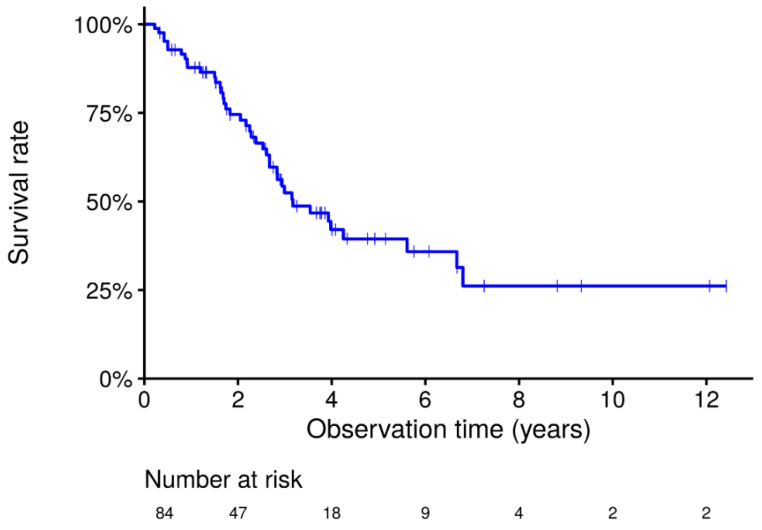
Kaplan-Meier survival curves in the total cohort of idiopathic PPFE. Overall survival of the 84 patients with idiopathic PPFE. Survival at 5 and 10 years was 38.5% and 26.1%, respectively.

The 5-year and 10-year survival rates were approximately 38% and 26%, respectively, which suggested that the survival of the total idiopathic PPFE population in this cohort was poor (Figure 1).

A multivariate Cox proportional hazards model showed that being male (*HR* = 6.594, 95% CI: 2.484 to 17.505) and having dyspnea (*HR* = 4.484, 95% CI: 1.616 to 12.421) were risk factors for all-cause mortality (Table 2).

### 3.2. Cluster Analysis

Among the patients with data for each variable, four clusters were identified. There were unique differences in the clinical characteristics of the four clusters. The characteristics of the patients in cluster 1 (*n* = 25, 30%) were as follows: male, non-smoker, non-symptomatic, coexistent with a certain degree of lower lobe lesions with a non-UIP-like pattern, with the highest baseline FVC and DLCO. The patients in cluster 2 (*n* = 26, 31%) were female, approximately 100% were non-smokers, relatively symptomatic, and coexistent with lesser lower lobe lesions than in cluster 1. The patients in cluster 3 (*n* = 18, 21%) were male, symptomatic smokers, coexistent with lower lobe lesions with a more UIP-like pattern, with the lowest baseline FVC. The patients in cluster 4 (*n* = 15, 18%) were also male, youngest among the clusters, asymptomatic, and without lower lobe lesions. Moreover, many patients in cluster 4 had a history of pneumothorax (Table 3).

**Figure 2:**
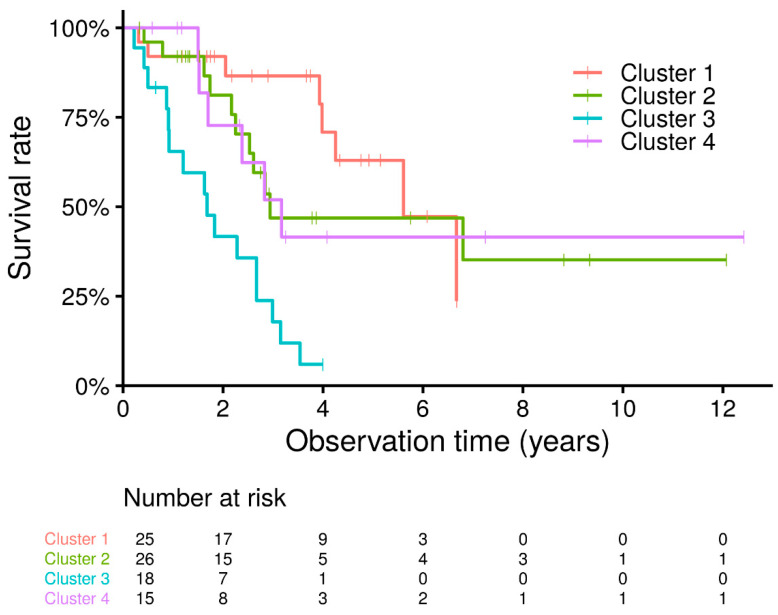
Kaplan–Meier survival curves according to idiopathic PPFE phenotypes. Survival of cluster 3 is significantly worse than that of the other clusters (cluster 3 vs. cluster 1, *p* < 0.001; vs. cluster 2, *p* = 0.0041; vs. cluster 4, *p* = 0.0155). No differences were found between clusters 1, 2, and 4 (cluster 1 vs. cluster 2, *p* = 0.4206; vs. cluster 4, *p* = 0.3515; cluster 2 vs. cluster 4, *p* = 0.9774).

Survival analysis of idiopathic PPFE phenotype demonstrated that patients in cluster 3 had significantly worse survival rates than those in any of the other clusters (cluster 3 vs. cluster 1. *p* < 0.001; vs. cluster 2, *p* = 0.0041; vs. cluster 4, *p* = 0.0155). There was no difference between clusters 1, 2, and 4 (cluster 1 vs. cluster 2, *p* = 0.4206; vs. cluster 4, *p* = 0.3515; cluster 2 vs. cluster 4, *p* = 0.9774) (Figure 2).

## 4. Discussion

As previously reported [11,12,13], we found an overall poor prognosis in patients with idiopathic PPFE. Prognostic factors in this condition have also been investigated. In this study, multivariate analysis revealed that male sex and dyspnea were independent prognostic factors. Consistent with these data, we had previously found that male sex and low elector spinae muscle attenuation, as determined via a CT scan, were independent poor prognostic factors in patients with idiopathic PPFE [14]. Khiroya et al. also reported that only male sex was correlated with increased mortality risk in 43 idiopathic PPFE cases [15]. Dyspnea was also a prognostic factor in this study. Dyspnea is seen in several pulmonary diseases and is used to assess the quality of life, disease severity, and prognosis. In IPF, the dyspnea score at baseline and change in score at six and 12 months have been shown to be significant and independent predictors of survival after adjustment for disease severity by physiologic parameters [16,17]. There have been no reports of idiopathic PPFE. Due to the fact that dyspnea is a common symptom in patients with idiopathic PPFE, future studies may reveal the significance of dyspnea in idiopathic PPFE.

We used cluster analysis to further study prognostic factors in idiopathic PPFE and identified clusters with distinct clinical and radiologic features and different prognoses. Eventually, we found that patients in cluster 3 had a significantly poorer prognosis than those in the other clusters. As expected, cluster 3 included male patients with dyspnea. Interestingly, those patients also had lower lobe ILD, particularly lower lobe UIP-like patterns, showing that the cluster has a clinically more ‘IPF-like’ phenotype.

IPF and PPFE are both reported to be progressive and devastating diseases among IIPs [8,11]. In some patients with IPF, PPFE lesions are observed in the upper lobe. It is sometimes difficult and somewhat arbitrary to differentiate and diagnose PPFE in patients with IPF [18], depending on the predominance of these lesions in the individual case. Moreover, it remains controversial whether the presence of PPFE or radiologic findings suggests that PPFE may affect the outcome. Oda et al. reported that the survival time of PPFE with the UIP pattern tended to be shorter than that of IPF [19]. However, Lee et al. recently reported radiologic findings suggesting that PPFE was an independent risk factor for pneumothorax or pneumomediastinum, except for mortality in patients with IPF [20]. Meanwhile, in our previous study with a small cohort, there were no significant differences in prognosis between patients with idiopathic PPFE who presented with lower-lobe UIP/possible UIP pattern and those without. However, later with different cohorts, PPFE patients with a UIP pattern in the lower lobe have been reported to have a significantly worse prognosis than those without lower lobe lesions or with non-UIP patterns [21,22]. Our unique cluster analysis results showed that the IPF-like PPFE phenotype had a worse prognosis than other PPFE phenotypes, suggesting that the IPF-like PPFE phenotype might be a distinct phenotype.

In this study, other different points of view, UIP-like pattern included UIP, probable UIP, and indeterminate for UIP patterns, as they are defined radiologically [8]. This may suggest that having some degree of morphological UIP features with appropriate clinical characteristics has a worse prognosis in idiopathic PPFE. No treatment has been shown to be effective for the management of idiopathic PPFE [23]. Meanwhile, antifibrotic drugs are available for reducing forced vital capacity (FVC) decline in progressive fibrosing interstitial lung disease [24], as well as IPF [25]. It might be challenging, but it is valuable to study the efficacy of antifibrotics for IPF-like PPFE phenotype.

Furthermore, except for cluster 3, the results of the cluster analysis might be clinically meaningful for managing patients. For instance, patients in cluster 4 had a significant history of pneumothorax. Although pneumothorax is an important event for all PPFE patients, relatively younger male patients without lower lobe lesions might require careful observation during the disease course. Some patients in clusters 1, 2, and 4 appeared to have longer survival than those in cluster 3. It has also been reported that PPFE patients who have a stable disease at first may show a sudden progressive course several years later [3]. The ‘silent’ period of the disease course in PPFE patients should be investigated in the future.

This study had several limitations. First, this was a retrospective study. Second, a small number of patients were included because of the rarity of the disease. Third, this study used one series of criteria among several proposed clinical diagnostic criteria [7,26,27]. Finally, the present study evaluated the prognosis in patients with idiopathic PPFE; however, the detailed clinical course of idiopathic PPFE, such as insidious spirometric decline before idiopathic PPFE diagnosis, was not assessed. Further studies are required to examine these issues.

## 5. Conclusions

Among the diverse progressive idiopathic PPFE patients, cluster analysis using each characteristic identified four distinct clinical phenotypes that might predict survival outcomes. The ‘IPF-like’ PPFE phenotype had a significantly poor prognosis than the other clusters, suggesting that it might be a distinctive phenotype in idiopathic PPFE.

## Figures and Tables

**Table 1:** Clinical Characteristics of the patients.

Number of patients	84
Year-patients	245.9
Age	69.0 [14.8, 18.4]
Sex Male/Female, *n* (%)	54 (64.3)/30 (35.7)
Current or former smoker, *n* (%)	31 (36.9)
Cough, *n* (%)	31 (36.9)
Dyspnea, *n* (%)	40 (47.6)
BMI	17.3 [14.7, 18.5]
Laboratory testing: KL-6, U/mL	472.0 [361.0, 621.5]
SP-D, ng/mL	180.0 [133.3, 262.5]
PaO2, Torr	80.0 [72.2, 89.0]
PaCO2, Torr	46.6 [41.8, 49.2]
Pulmonary function: % FVC, %	60.5 [47.0, 77.9]
FEV1.0/FVC, %	95.9 [90.1, 100.0]
% DLCO, %	77.7 [68.1, 102.5]
RV/TLC, %	48.2 [42.8, 59.2]

Data are presented as *n* (%) or median (interquartile range). *n* = number; BMI: body mass index; KL-6: Krebs von den Lungen-6; SP-D: Surfactant protein-D; DLCO: diffusing capacity of the lung for carbon monoxide; FVC: forced vital capacity; FEV1.0: Forced Expiratory Volume; RV: residual volume; TLC: total lung capacity.

**Table 2:** Multivariate Cox proportional hazard model in idiopathic PPFE patients for the mortality risk.

Variable	*HR*	95% CI lower	95% CI upper	*p*-Value
Age	1.035	0.991	1.079	0.117
Sex, Male	6.594	2.484	17.505	<0.001
Dyspnea	4.480	1.616	12.421	0.004
FVC, %	0.982	0.959	1.007	0.156

**Table 3:** Differences in clinical characteristics between the four clusters.

Variable	Cluster 1	Cluster 2	Cluster 3	Cluster 4	*p*-Value
Number of patients	25	26	18	15	
Year-patients	83.9	84.0	32.1	45.8	
Age	69 [61.0, 75.0]	72.5 [65.0, 79.0]	71.5 [67.5, 73.8]	64 [58.0, 69.0]	0.061
Sex, male	21 (84.0)	3 (11.5)	18 (100)	12 (80.0)	<0.001
Smoking	7 (28.0)	0 (0.0)	15 (83.3)	9 (60.0)	<0.001
Cough	9 (36.0)	8 (30.8)	4 (22.2)	10 (66.7)	0.06
Dyspnea	0 (0.0)	18 (69.2)	18 (100.0)	4 (26.7)	<0.001
Fine crackles	6 (24.0%)	8 (30.8)	12 (66.7)	1 (6.7)	0.002
BMI	17.9 [16.1, 19.9]	15.2 [14.0, 17.3]	17.6 [14.8, 18.4]	16.6 [15.1, 18.6]	0.022
pFVC	83 [60.5, 90.3]	52.6 [37.5, 60.2]	48.7 [40.0, 62.4]	74.7 [59.1, 78.5]	<0.001
FEV1/FVC	91.6 [88.9, 97.0]	96.1 [93.3, 100.0]	100 [94.6, 100.0]	97.8 [95.0, 100.0]	0.02
RV/TLC	43.8 [37.9, 46.7]	57.6 [46.9, 62.4]	54.1 [46.4, 59.5]	50 [43.5, 53.5]	0.056
pDLco	98.1 [92.5, 115.0]	75.5 [69.2, 86.8]	68.7 [58.4, 83.2]	77.7 [68.7, 109.8]	0.054
Alb	4 [3.8, 4.1]	4 [3.8, 4.4]	3.8 [3.3, 4.1]	4.2 [4.0, 4.5]	0.06
LDH	182.5 [174.8, 209.5]	214 [188.2, 246.8]	199 [189.0, 226.8]	194.5 [162.8, 210.0]	0.03
KL6	392 [331.0, 486.2]	525 [471.2, 637.8]	569 [410.0, 929.0]	389 [358.0, 478.1]	0.003
SPD	149.6 [108.5, 209.0]	173 [133.0, 258.0]	252 [204.0, 377.0]	179 [125.0, 223.0]	0.004
CT: lower lobe	20 (80.0)	17 (65.4)	17 (94.4)	3 (20.0)	<0.001
CT: UIP like	12 (48.0)	12 (46.2)	13 (72.2)	3 (20.0)	0.029
Pneumothorax	5 (20.0)	6 (23.1)	4 (22.2)	12 (80.0)	<0.001

Data are presented as *n* (%) or median (interquartile range). BMI: body mass index; FVC: forced vital capacity; FEV: forced expiratory volume in 1 s; RV: residual volume; TLC: total lung capacity; DLCO: diffusing capacity of the lung for carbon monoxide; LDH: lactate dehydrogenase; KL-6: Krebs von den Lungen-6; SP-D: surfactant protein-D; UIP: usual interstitial pneumonia.

## Data Availability

The data presented on this study are available from the corresponding author upon reasonable request.

## Supplementary Materials

The following are available online at https://www.mdpi.com/article/10.3390/jcm10071498/s1, Table S1: The data for the other variables.
