# Supplementary material for: Prognostic and Clinical Value of Cluster Analysis in Idiopathic Pleuroparenchymal Fibroelastosis Phenotypes
Source: J Clin Med. 2021 Apr 4;10(7):1498. doi: 10.3390/jcm10071498 (PMC8038478; doi:10.3390/jcm10071498)
Supplement: Supplementary file 1 [file jcm-10-01498-s001.pdf]

Journal of
Clinical Medicine

MDPI

Article

Supplementary Material: Prognostic and Clinical Value of Cluster Analysis in Idiopathic Pleuroparenchymal Fibroelastosis Phenotypes

Yutaro Nakamura, Kazutaka Mori, Yasunori Enomoto, Masato Kono, Hiromitsu Sumikawa, Takeshi Johkoh, Thomas V. Colby, Hideki Yasui, Hironao Hozumi, Masato Karayama, Yuzo Suzuki, Kazuki Furuhashi, Tomoyuki Fujisawa, Noriyuki Enomoto, Naoki Inui, Yusuke Kaida, Koshi Yokomura, Naoki Koshimizu, Mikio Toyoshima, Shiro Imokawa, Takashi Yamada, Toshihiro Shirai, Hidenori Nakamura, Hiroshi Hayakawa and Takafumi Suda

Table S1. The data for the other variables.

Dust exposure, *n* (%)	15 (17.9)
Malignancy, *n* (%)	12 (14.3)
Pneumothorax, *n* (%)	27 (32.1)
GI disease, *n* (%)	16 (19.0)
Autoantibody, *n* (%)	7 (8.33)
Fine crackles, *n* (%)	27 (32.1)
CT, lower lobe lesion, *n* (%)	57 (67.5)
Alb, g/dL	4.00 [3.70, 4.30]
LDH, IU/L	197.0 [181.0, 230.7]

Data are presented as *n* (%) or median (interquartile range). *n* = number; GI: Gastrointestinal; Alb: Albumine; LDH: lactate dehydrogenase.
